# Hypervirulent *Klebsiella pneumoniae* in a South African tertiary hospital—Clinical profile, genetic determinants, and virulence in *Caenorhabditis elegans*

**DOI:** 10.3389/fmicb.2024.1385724

**Published:** 2024-05-23

**Authors:** Likhona Dingiswayo, Olusesan Adeyemi Adelabu, Emmanuel Arko-Cobbah, Carolina Pohl, Nthabiseng Zelda Mokoena, Morne Du Plessis, Jolly Musoke

**Affiliations:** ^1^Department of Medical Microbiology, School of Pathology, Faculty of Health Sciences, University of the Free State, Bloemfontein, South Africa; ^2^Department of Surgery, School of Clinical Medicine, Faculty of Health Sciences, University of the Free State, Bloemfontein, South Africa; ^3^Department of Microbiology and Biochemistry, Faculty of Natural and Agricultural Sciences, University of the Free State, Bloemfontein, South Africa; ^4^Department of Genetics, Faculty of Natural and Agricultural Sciences, University of the Free State, Bloemfontein, South Africa; ^5^National Health Laboratory Service, Department of Medical Microbiology, Universitas Academic Hospital, Bloemfontein, South Africa

**Keywords:** hypervirulent, *Klebsiella pneumoniae*, virulence, *Caenorhabditis elegans*, aerobactin, capsule, serotype

## Abstract

**Introduction:**

A distinct strain of *Klebsiella pneumoniae* (*K. pneumoniae*) referred to as hypervirulent (hvKp) is associated with invasive infections such as pyogenic liver abscess in young and healthy individuals. In South Africa, limited information about the prevalence and virulence of this hvKp strain is available. The aim of this study was to determine the prevalence of hvKp and virulence-associated factors in *K. pneumoniae* isolates from one of the largest tertiary hospitals in a South African province.

**Methods:**

A total of 74 *K. pneumoniae* isolates were received from Pelonomi Tertiary Hospital National Health Laboratory Service (NHLS), Bloemfontein. Virulence-associated genes (*rmpA*, capsule serotype K1/K2, *iroB* and *irp2*) were screened using Polymerase Chain Reaction (PCR). The *iutA* (aerobactin transporter) gene was used as a primary biomarker of hvKp. The extracted DNAs were sequenced using the next-generation sequencing pipeline and the curated sequences were used for phylogeny analyses using appropriate bioinformatic tools. The virulence of hvKp vs. classical *Klebsiella pneumoniae* (cKp) was investigated using the *Caenorhabditis elegans* nematode model.

**Results:**

Nine (12.2%) isolates were identified as hvKp. Moreover, hvKp was significantly (*p* < 0.05) more virulent *in vivo* in *Caenorhabditis elegans* relative to cKp. The virulence-associated genes [*rmpA, iroB*, hypermucoviscous phenotype *(hmv)* phenotype and capsule K1/K2] were significantly (*p* < 0.05) associated with hvKp. A homology search of the curated sequences revealed a high percentage of identity between 99.8 and 100% with other homologous *iutA* gene sequences of other hvKp in the GenBank.

**Conclusion:**

Findings from this study confirm the presence of hvKp in a large tertiary hospital in central South Africa. However, the low prevalence and mild to moderate clinical presentation of infected patients suggest a marginal threat to public health. Further studies in different settings are required to establish the true potential impact of hvKp in developing countries.

## 1 Introduction

The hypervirulent *Klebsiella pneumoniae* (hvKp), a causative agent of fulminant and invasive diseases relating to community-acquired *pneumoniae* (CAP), capable of bearing plasmids of hypervirulence or carbapenem resistance, is recognized as another circulating pathotype in addition to classical *K. pneumoniae* (cKp), which is a frequent pathogenic agent relating to hospital-acquired *pneumoniae* (HAP), considered to have limited virulence capability compared to hvKp (Karampatakis et al., 2023). This pathotype is associated with high pathogenicity and mortality and was initially identified in Taiwan in the mid-1980s (Russo and Marr, 2019; Wang et al., 2019). An increasing number of reports across the globe has indicated the geographical spread of hvKp (Shon et al., 2013). Liu et al. (2014) and Nahavandinejad and Asadpour (2017) reported an incidence of 31.4% (22/70) and 33.8% (22/65) of hypervirulent cases in China and Northern Iran, respectively. In South Africa (SA), limited studies on hvKp have been conducted, with only referred reports (collaborative studies conducted abroad that included samples collected in SA). Consequently, little is known about the true prevalence, clinical significance and presentation of hvKp infections.

Previously, hvKp was clinically associated with the ability to cause invasive pyogenic liver abscess (PLA) in a host (Pomakova et al., 2012; Russo and Marr, 2019). However, this pathotype has been clinically reported to cause community-acquired infections (CAI) which spreads to the eyes (endophthalmitis) and brain (meningitis) (Ullmann and Podschun, 1998; Pomakova et al., 2012; Russo et al., 2015; Paczosa and Mecsas, 2016). Unlike cKp infections, hvKp infections usually affect young and healthy individuals (Russo and Marr, 2019; Choby et al., 2020). However, current studies have reported the intrusion of hvKp in the healthcare settings (Li et al., 2018; Lan et al., 2019; Mukherjee et al., 2021). The mortality rate of hvKp infections ranges between 3 and 32% in healthy individuals in the community, suggesting that hvKp is a variant of concern and should be monitored (Han, 1995; Wang et al., 1998; Ko et al., 2002; Fang et al., 2007; Pomakova et al., 2012). In China, the incidence rate of hvKp infections has been reported to be ~74%, and a high mortality rate of 60%, with community transmission being further promoted via colonization of gastrointestinal system (Choby et al., 2020; Su et al., 2021). Several genetic determinants are associated with hypervirulence that distinguish hvKp from cKp (Wu et al., 2017; Lam et al., 2018a,b; Wyres et al., 2020).

The most common feature of hvKp is the increased production of a capsule, mainly capsule K1 and K2 (Ullmann and Podschun, 1998; Remya et al., 2018; Russo et al., 2018). A relationship between the presence of capsule type and serotypes (sequence type) has been reported, which has shed some additional light in the virulence of hvKp (Conlan et al., 2012; Hyun et al., 2019). The sequence type (ST) 23 is believed to be associated with K1 capsule type, while distinct STs (e.g., ST 65, ST 86, ST 375, and ST 380) are associated with K2 (Follador et al., 2016; Catalán-Nájera et al., 2017; Martin and Bachman, 2018). Iron chelators (siderophores), such as aerobactin and salmochelin, have also been reported to be associated with increased virulence in hvKp (Wu et al., 2017; Lam et al., 2018a,b; Wyres et al., 2020). These genetic determinants are located on large virulence plasmids along with the regulator of mucoid phenotype A (*rmpA*, and *rmpA2*) that enhances the chromosomal capsule polysaccharide (CPS) (Choby et al., 2020). The result of enhanced expression of CPS is a hyper-capsule, a phenomenon known as a hypermucoviscous phenotype detected by a string test (Russo and Marr, 2019; Choby et al., 2020).

The occurrence of hvKp varies and on average ranged between 12 and 45% in regions of China including Hong Kong, Beijing, Changsha, and Anhui (Zhang et al., 2016; Liu et al., 2018, 2019; Lan et al., 2019). However, in developing countries, this variant is often unidentified. Therefore, this study aimed to characterize *K. pneumoniae* isolates through clinical, molecular, and genomic studies to provide a broader knowledge about the newly emerging hvKp, and whether it is a public concern in developing countries similar to what is seen globally.

## 2 Materials and methods

### 2.1 Study design and location

This was a descriptive, cross-sectional study using isolates previously identified as *K. pneumoniae* routinely using the VITEK^®^ 2 system (bioMérieux, France) by the National Health Laboratory Service (NHLS), Pelonomi Tertiary Hospital, in Bloemfontein, Free State Province, South Africa. Over a period of 12 months (September 2020 to October 2021), a total of 74 *K. pneumoniae* isolates were obtained from the laboratory. For patient confidentiality, all patient names were anonymized and a study number was assigned to each isolate. The selected *K. pneumoniae* isolates were from different wards in the hospital, including the medical ward, maternity, casualty, multidisciplinary intensive care unit (MICU), trauma unit, outpatient department (OPD) and neonatal unit. Furthermore, these isolates were obtained from different sample types including blood cultures, tracheal aspirate, urine, and sputum.

### 2.2 Patient demographics and antimicrobial susceptibility profiles

The laboratory database, NHLS TrakCare Lab WebView system, was used to extract antibiotic susceptibility profiles for each isolate. Patients demographic information and clinical data, including type of sample taken, age, gender, and the day the sample was taken, were also obtained from the NHLS TrakCare WebView system. The date of admission for each patient and clinical presentation were extracted from the Meditech Medical Record System. Hospital-acquired infections were defined as *K. pneumoniae* infection acquired by patients 48 h after hospitalization, while community-acquired infections were defined as infection occurring 48 h prior to hospitalization. Mild symptoms were treated on an outpatient basis. Moderate to severe disease was defined as patients presenting with organ impairment due to *K. pneumoniae* infection were patients who were admitted to the intensive care unit (ICU), while mild infections were patients who were admitted to normal wards or outpatients.

### 2.3 String test for detection of hypermucoviscous phenotypes

All the *K. pneumoniae* isolates obtained (*n* = 74) were sub-cultured onto 5% blood agar and incubated at 37°C for 24 h. A string test was performed on all isolates as described by Guo et al. (2017). A colony length of ≥5 mm was defined as positive for the hypermucoviscous (hmv) phenotype.

### 2.4 Molecular detection of *Klebsiella pneumoniae* strains

Crude DNA was extracted from all 74 *K. pneumoniae* isolates. Briefly, samples were heated at 95°C for 30 min and frozen at −80°C for 30 min. The samples were then centrifuged at 13 000 rpm for 10 min and the supernatant was used after centrifugation. A total of six separate conventional single-plex PCR reactions were performed on all isolates. The target sites *iuc* transporter (*iutA*), *rmpA*, capsule K1/K2 (*magA*/*k2A*, respectively), yersiniabactin (*irp2*) and *iroB* siderophores were used (Table 1).

**Table 1:** The list of primers used for detection of K1/K2 (*magA*/*k2A*) serotypes*, rmpA, iutA, iroB*, and *irp2*.

**Gene**	**Primer sequence (5^′^-3^′^)**	**Band size (bp)**	**References**
*magA*	F-5′-GGTGCTCTTTACATCATTGC-3′	1,283	Compain et al., 2014; Remya et al., 2018
	R-3′-GCAATGGCCATTTGCGTTAG-5′		
*k2A*	F-5′-CAACCATGGTGGTCGATTAG-3′	532	Remya et al., 2018
	R-3′-TGGTAGCCATATCCCTTTGG-5′		
*rmpA*	F-5′-CATAAGAGTATTGGTTGACAG-3′	461	Compain et al., 2014; Remya et al., 2018
	R-3′-CTTGCATGAGCCATCTTTCA-5′		
*iutA*	F-5′-GGGAAAGGCTTCTCTGCCAT-3′	920	Compain et al., 2014
	R-3′-TTATTCGCCACCACGCTCTT-5′		
*iroB*	F-5′-ATCTCATCATCTACCCTCCGCTC-3′	235	Russo et al., 2018
	R-3′-GGTTCGCCGTCGTTTTCAA-5′		
*irp2*	F-5′-GCTACAATGGGACAGCAACGAC-3′	230	Russo et al., 2018
	R-3′-GCAGAGCGATACGGAAAATGC-5′		

The PCR amplicons were separated using 1.5% SeaKem™ LE Agarose (Lonza™, USA) and the nucleic acid stain gelRed (Biotum GelRed™, Australia), as per the manufacturer's instructions, for 40 min at 100 volts (V). The gel was viewed using the Geldoc™ EZ system (Bio-Rad, USA) for the detection of specific band sizes. Two reference molecular markers were used, including 200 and 500 + 100 bp (Thermo Fisher Scientific, USA). The hvKp was defined as isolates bearing the *iuc* transport genetic determinant, *iutA* (Russo et al., 2018).

### 2.5 *In vivo Caenorhabditis elegans* killing assay

Seven *Klebsiella pneumoniae* (*K. pneumoniae*) representatives of different spectra of hypervirulent *Klebsiella pneumonia*e (hvKp) genetic determinants and classical *Klebsiella pneumoniae* (cKp) were selected for *in vivo* assay (shown in Figure 3A). Summarily, the selection criteria for this assay included two (2) cKp that possessed *irp*2 (Isolate 54) and K2 (Isolate 60) gene, respectively. Five hvKp that possessed all five genes with K1/K2 (Isolate 1, 5, and 17, respectively), isolate 24 (possess four virulent genes/K1), isolate 51 (had only *irp*2 gene). The *Caenorhabditis elegans* (*C. elegans*) *glp-4; sek-1* hermaphrodites used in this study was obtained from the *Caenorhabditis* Genetic Center, College of Biological Sciences, University of Minnesota, USA. *Escherichia coli* OP50 was used as a food source for the nematodes (Brenner, 1974). The *C. elegans* were infected with the seven selected strains of *K. pneumoniae* according to a modified protocol described by Kamaladevi and Balamurugan (2016). Briefly, after moving Synchronized L4 *C. elegans* to brain heart infusion (BHI) plates (EMD Millipore, Germany) seeded with selected strains of *K. pneumoniae*, the plates were incubated at 25°C for 4 h, followed by washing of *C. elegans* with 9 ml of M9 buffer (Sigma-Aldrich, USA). The plates were incubated at 25°C and the nematodes were scored daily as alive or dead. The *C. elegans* were regarded as dead when no movement in response to prickling mechanical stimulation was observed and were removed from the media and discarded. The experiment was done in triplicate.

### 2.6 DNA library preparation, sequencing, editing, genome assembly and phylogeny analysis

The extracted DNA of five *K. pneumoniae* was sent for sequencing at the Agricultural Research Council (ARC), Pretoria, South Africa. The selected isolates were representatives of both hvKp (*n* = 4), and cKp (*n* = 1) and their selection was based on the *C. elegans* killing assay results. To construct a DNA library for the sequencing of the whole genome of *K. pneumoniae*, a Nextera^®^ XT library preparation kit (Illumina^®^, San Diego, CA, USA) was used to construct a DNA library adhering to the manufacturer's procedures. This involved the fragmentation of DNA followed by the addition of dual barcodes to the DNA fragments. For the purification of the barcoded libraries, Agencourt AMPure magnetic beads (Beckman Coulter, Indianapolis, Indiana, USA) were utilized and the selection of an average insert of 300 bp (range 200–400 bp) was carried out simultaneously. In addition, validation of the library and quantification were conducted prior to sequencing using a 2100 Bioanalyzer platform (Agilent Technologies, Santa Clara, CA, USA) and the Qubit™ 3.0 fluorometer (Invitrogen, Carlsbad, CA, USA), respectively. This was followed by the pooling of the validated and quantified libraries, and the whole genome sequencing was executed, using a 5% PhiX DNA control spike-in, on an Illumina^®^ MiSeq platform with using the Illumina MiSeq platform using a v3 standard sequencing kit (Illumina^®^, San Diego, CA, USA) for 500 cycles.

The raw sequence files (fastq format) were retrieved from Illumina Miseq instrument.

Quality control of sequences was performed using FastQC (v.0.12.1; https://www.bioinformatics.babraham.ac.uk/projects/fastqc/), whereafter trimming and removal of barcodes was achieved with BBDuk which is a component of the BBTools package (https://sourceforge.net/projects/bbmap/). Sequence assembly was performed with Spades (v.3.15.5; Prjibelski et al., 2020). The assembled genomes were assessed for completeness using QUAST (https://github.com/ablab/quast). An additional analysis of genome completeness was performed using BUSCO (v.5.4.7; https://busco.ezlab.org/). The identification of putative virulence genes was achieved through a BLAST comparison of assembled contigs against a database of representative sequences for all virulence genes as identified from the Institut Pasteur *Klebsiella pneumoniae* virulence genes scheme (https://bigsdb.pasteur.fr/cgi-bin/bigsdb/bigsdb.pl?db=pubmlst_klebsiella_seqdef&page=downloadAlleles). Phylogeny analysis was conducted using a 1,000 replicate bootstrapping and the distance-based neighbor-joining algorithm method as implemented in Mega 11 software was adopted to compute the evolutionary distances of the aligned sequences (Tamura et al., 2021). Annotation and plasmid visualization of the plasmid were conducted using SnapGene^®^ software version 7.1.1 (available at www.snapgene.com).

### 2.7 Statistical analysis

Genetic determinants of these isolates were analyzed using logistic regression in MS Excel (version 2016) to identify variables associated with hvKp. The presence of *iutA* was used as an independent variable for defining hvKp. Statistical significance between the virulence levels of distinct strains (hvKp, cKp and *E. coli* OP50) was analyzed using the online application for survival analysis version 2 (OASIS 2) as described by Han et al. (2016). All experiments performed in the *C. elegans* killing assay were done in triplicate and were expressed as a mean with standard deviation. A *p*-value of < 0.05 was considered statistically significant using logistic regression in MS Excel.

### 2.8 Ethical considerations

Ethics approval to conduct the research was obtained from the Health Sciences Research Ethics Committee (HSREC) of the University of the Free State in Bloemfontein, South Africa (UFS-HSD2020/1579/2302) and Environmental and Biosafety Research Ethics Committee (UFS-ESD2020/0148). Further approval from the Free State Department of Health was obtained.

## 3 Results

### 3.1 Patient demographics and genotypic biomarker

Hypervirulence was found in nine (12.2%) of the 74 *K. pneumoniae* isolates (Supplementary Figure 1). The average patient age in hypervirulent *Klebsiella pneumoniae* (hvKp) strains was 35 years (19–57 years) while cKp was 34 years (Newborn–85).

The Clinical presentations and antimicrobial profiles of hvKp are summarized in Tables 2, 3 respectively. The antimicrobial susceptibility profile of cKp isolates is shown in the supplementary Table 2. The overall antimicrobial resistance of all isolates (*n* = 74) indicated extended-spectrum beta-lactamases (ESBLs) of (19%) whereby none of the hvKp were ESBL. Regarding Carbapenem-resistant *Enterobacteriaceae* (CRE), the overall incidence was 4%, none of which were hvKp.

**Table 2:** Clinical presentation of patients with hypervirulent *Klebsiella pneumoniae* (hvKp) infections.

**hvKp isolate number**	**Sample type**	**Source of infection**	**Spectrum of disease**
1	Blood culture	Infective endocarditis	Severe
2	Midstream urine	Urinary tract infection	Mild
5	Sputum	Pneumonia	Mild
8	Blood culture	Septicemia	Severe
17	Tracheal aspirate	Pneumonia	Moderate
24	Catheter urine	Urinary tract infection	Mild
40	Swab (superficial)	Wound sepsis	Mild
51	Midstream urine	Urinary tract infection	Mild
56	Sputum	Pneumonia	Severe

**Table 3:** The antimicrobial susceptibility profile of hypervirulent *Klebsiella pneumoniae* isolates.

**Antimicrobial drugs**	**Susceptibility results of hypervirulent** ***Klebsiella pneumoniae***								
	**1**	**2**	**5**	**8**	**17**	**24**	**40**	**51**	**56**
Ampicillin	R	R	R	R	R	R	R	R	R
Amoxicillin/clavulanic acid	S	S	S	N	S	S	S	S	S
Cefuroxime	S	S	S	S	S	S	S	S	S
Cefuroxime axetil	S	S	S	S	S	S	S	S	S
Cefotaxime	S	S	S	S	S	S	S	S	S
Gentamicin	S	S	S	S	S	S	S	S	S
Ciprofloxacin	S	S	S	S	S	S	S	S	S
Nitrofurantoin	S	I	S	N	S	I	S	N	I
Trimethoprim/ sulfamethoxazole	S	S	S	S	S	S	S	S	S
Ertapenem	S	S	S	S	S	S	S	S	S
Imipenem	S	S	S	S	S	S	S	S	S
Meropenem	S	S	S	S	S	S	S	S	S

S, susceptible; I, intermediate; R, resistant; N, antimicrobial testing was not performed.

**Figure 1:**
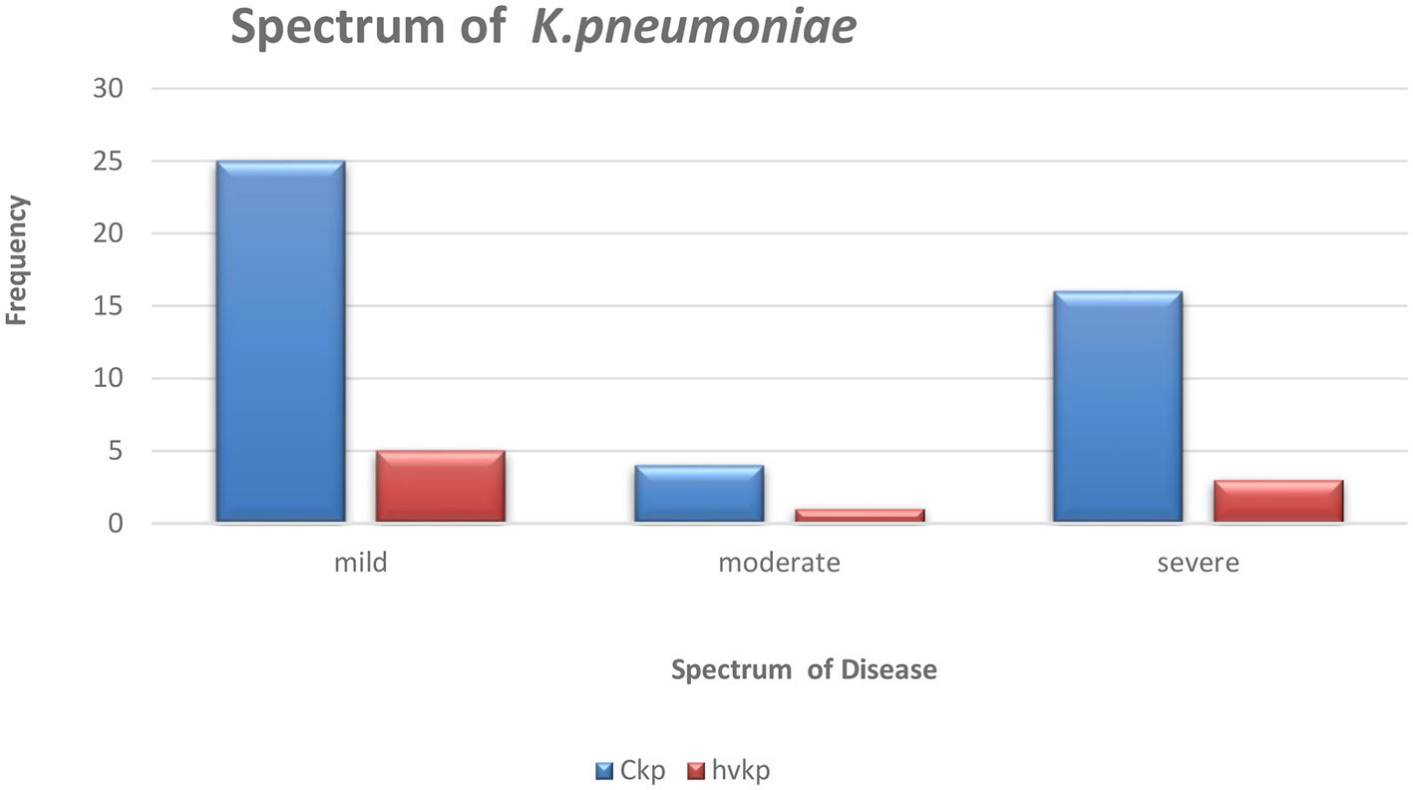
The graphical representation of the spectrum of *Klebsiella pneumoniae* disease amongst the studied isolates.

Hypermucoviscous phenotype (hmv) was found in 7/9 (78%) hvKp strains. An additional (1/64) classical *Klebsiella pneumoniae* (cKp) strain also possessed this phenotype. However, there was no statistical significance associated with the severity of the disease and *Klebsiella* pathotypes (Figure 1).

### 3.2 Virulence-associated factors of hvKp by PCR

The nine patients with hvKp in Pelonomi Tertiary Hospital varied regarding admission, as two patients came from the main medical ward and another two from the maternity ward. Other hvKp cases were from casualty, the multidisciplinary intensive care unit (MICU), the antenatal clinic and the out-patient department (OPD; *n* = 1 each). The presence of five genotypic traits [*iroB, rmpA*, K1 (*magA*), and K2 (*k2A*)] and a single phenotypic characteristic (hmv phenotype) were statistically associated *p* < 0.05) with hvKp (Table 4). Moreover, the virulence genes *rmpA*, K1 (*magA*), K2 (*k2A*) and *iroB* were detected in 88.9% (*n* = 8), 44.4% (*n* = 49), 55.6% (*n* = 5) and 88.9% (*n* = 8) hvKp strains, respectively. The presence of the *irp2* gene was not significantly associated with hvKp (*p* = 0.17) as 19 (29.2%) of the 65 cKp isolates possessed this gene. There was no statistically significant association between hvKp/cKp (*p* < 0.05) and the severity of disease. However, in 44.4% (*n* = 4/9) of infections caused by hvKp isolates, the patients had a moderate to severe spectrum of disease, whereas most patients (*n* = 40/65; 61.5%) with cKp infections had mild symptoms.

**Table 4:** Comparison of phenotypic and virulence-associated genes between hypervirulent *Klebsiella pneumoniae* (hvKp) and classical *K. pneumoniae* (cKp).

**Virulence-associated genes**	**hvKp (*n* = 9)**	**cKp (*n* = 65)**	*p*-value^*^
	***n*** **(%)**	***n*** **(%)**	
*iutA*	9 (100)	0 (0)	0.00
hmv phenotype (hypermucoviscous)	7 (77.8)	1 (1.5)	0.00
*rmpA*	8 (88.9)	0 (0)	0.00
*iroB*	8 (88.9)	0 (0)	0.00
Capsule K1 (*magA*)	4 (44.4)	0 (0)	0.00
Capsule K2 (*k2A*)	5 (55.6)	2 (3.1)	0.00
*irp2*	7 (77.8)	19 (29.2)	0.17

^*^A p-value of < 0.05 is considered to be statistically significant.

### 3.3 *Klebsiella pneumoniae* virulence in *Caenorhabditis elegans*

**Figure 2:**
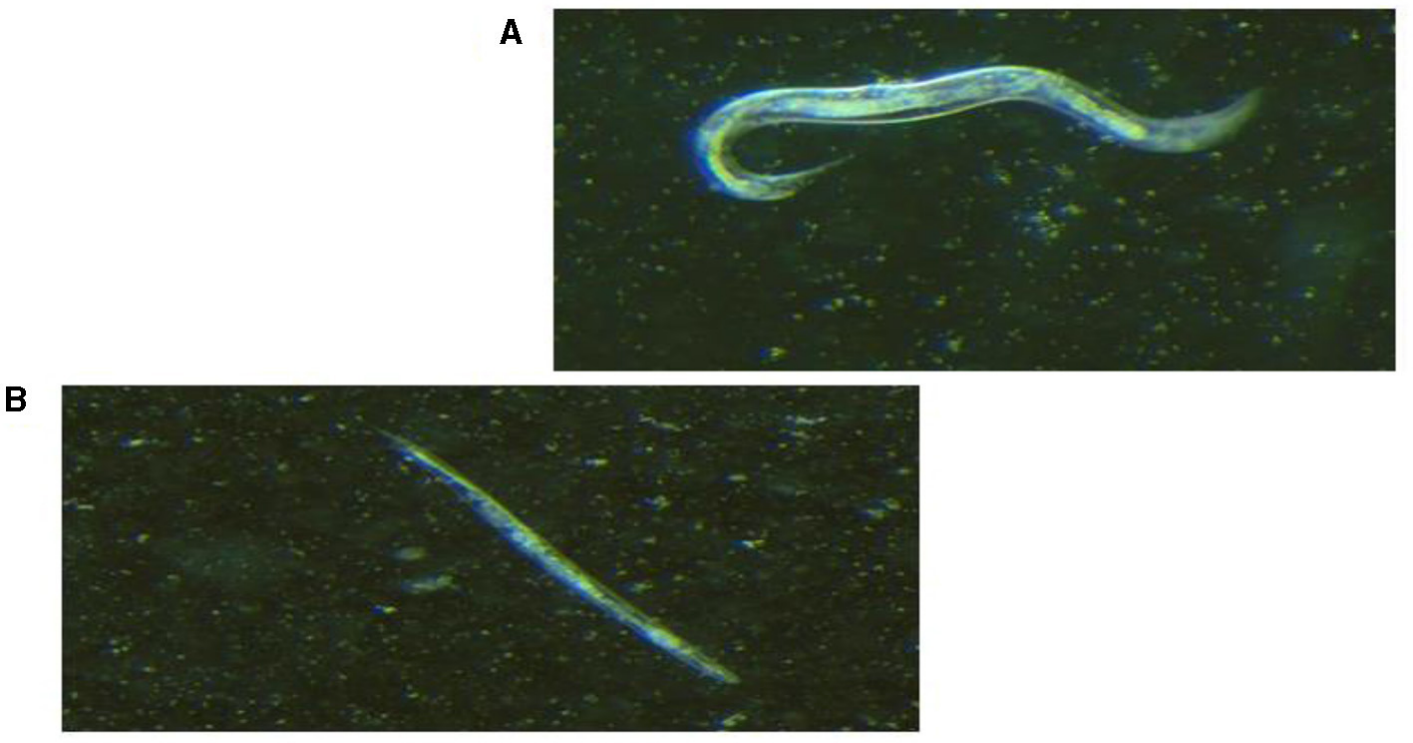
Light visualization of *Caenorhabditis elegans* under stereomicroscope ( × 1.28 magnification). **(A)** Live *C. elegans* that responded to prickling mechanical stimulation. **(B)** Dead, straight and rigid *C. elegans* that did not respond to prickling mechanical stimulation.

**Figure 3:**
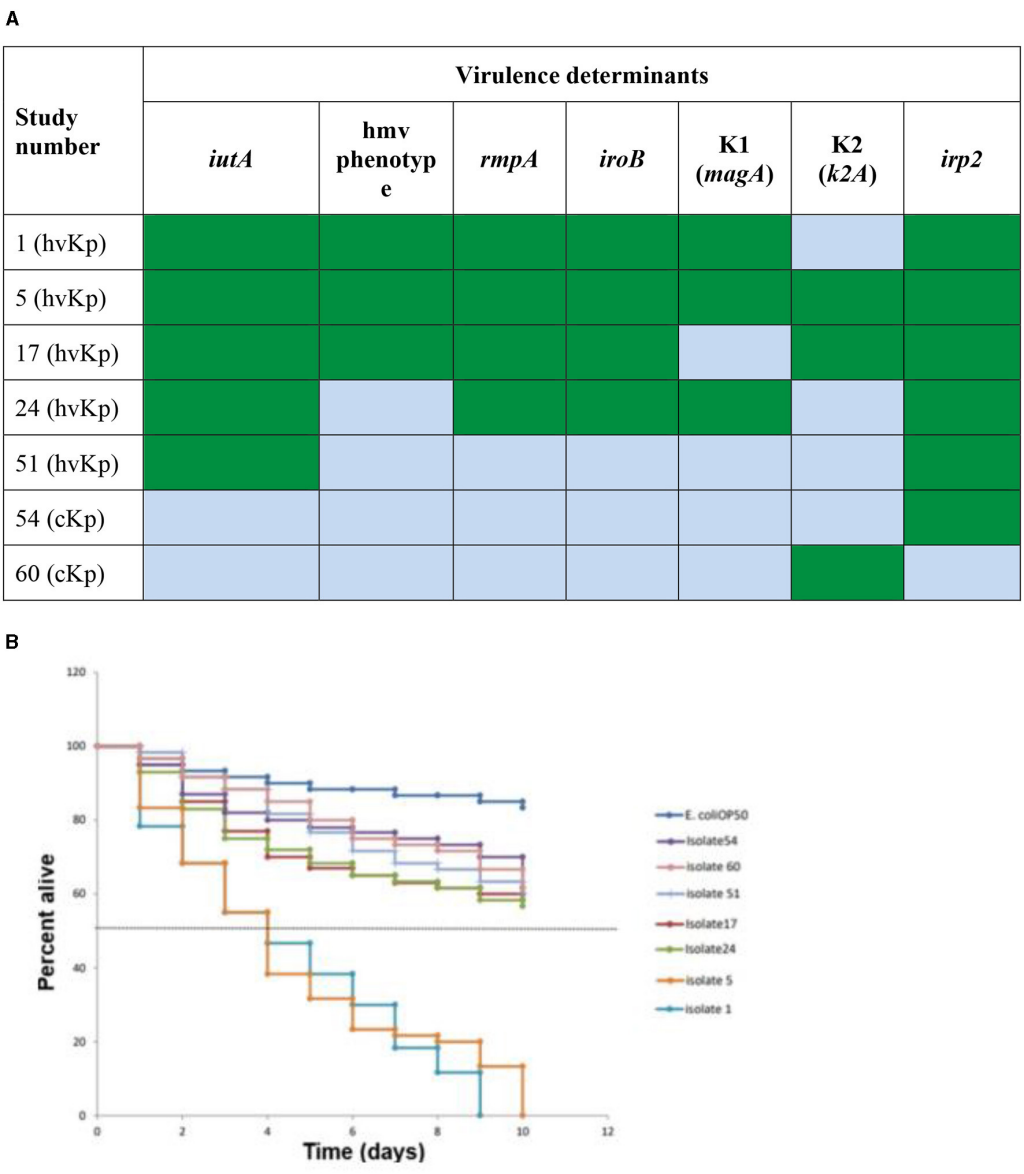
*In vivo Caenorhabditis elegans* infection model comparing hvKp and cKp. **(A)** Virulence genes present (green) or absent (light blue) in the strains selected for the infection model. Green: virulence determinant present; blue: virulence determinant absent. **(B)** The survival rate of *C. elegans* fed with different hvKp or cKp strains.

Live worms moved freely and responded to the prickling mechanical stimulation (movement when touched; Figure 2A), while dead *C. elegans* were rigid, straight, and floated on the media (Figure 2B). All seven selected *K. pneumoniae* strains (Figure 3B) showed a statistically significant (*p* < 0.05) increase in virulence compared to *C. elegans* exposed to *E. coli* OP50 only. The Two hvKp strains expressing all five genetic virulence markers showed enhanced virulence (*p* < 0.05), as none of the worms survived between day 9 and day 10, as shown in Figure 3B. In contrast, hvKp isolate 17 which had all five genetic determinants and belonged to K2 capsular type, had a reduced virulence compared to isolate 1 which had enhanced virulence (presence of five genetic determinants) and belonged to K1. There was no significant difference (*p* = 0.0795) in the virulence observed in a single *iutA*-positive hvKp (isolate 51) that also possessed the *irp2* gene.

### 3.4 *Klebsiella pneumoniae* sequence types and K-serotype

The population structure of the isolates was established by determining multi-locus sequence typing (MLST) and the K-serotype grouping (Larsen et al., 2012; Wyres et al., 2016). The sequences were identified for isolate 5, 7, 24, 51, and 54 as ST20, ST23, ST65, ST985, and ST3430, which is associated with the K loci groups KL1, KL2, KL28, KL39, and KL52, respectively, having a very high match confidence of ≥99% confidence to the reference locus, with no expected genes missing and no unexpected locus genes.

### 3.5 Genome sequence analysis

**Figure 4:**
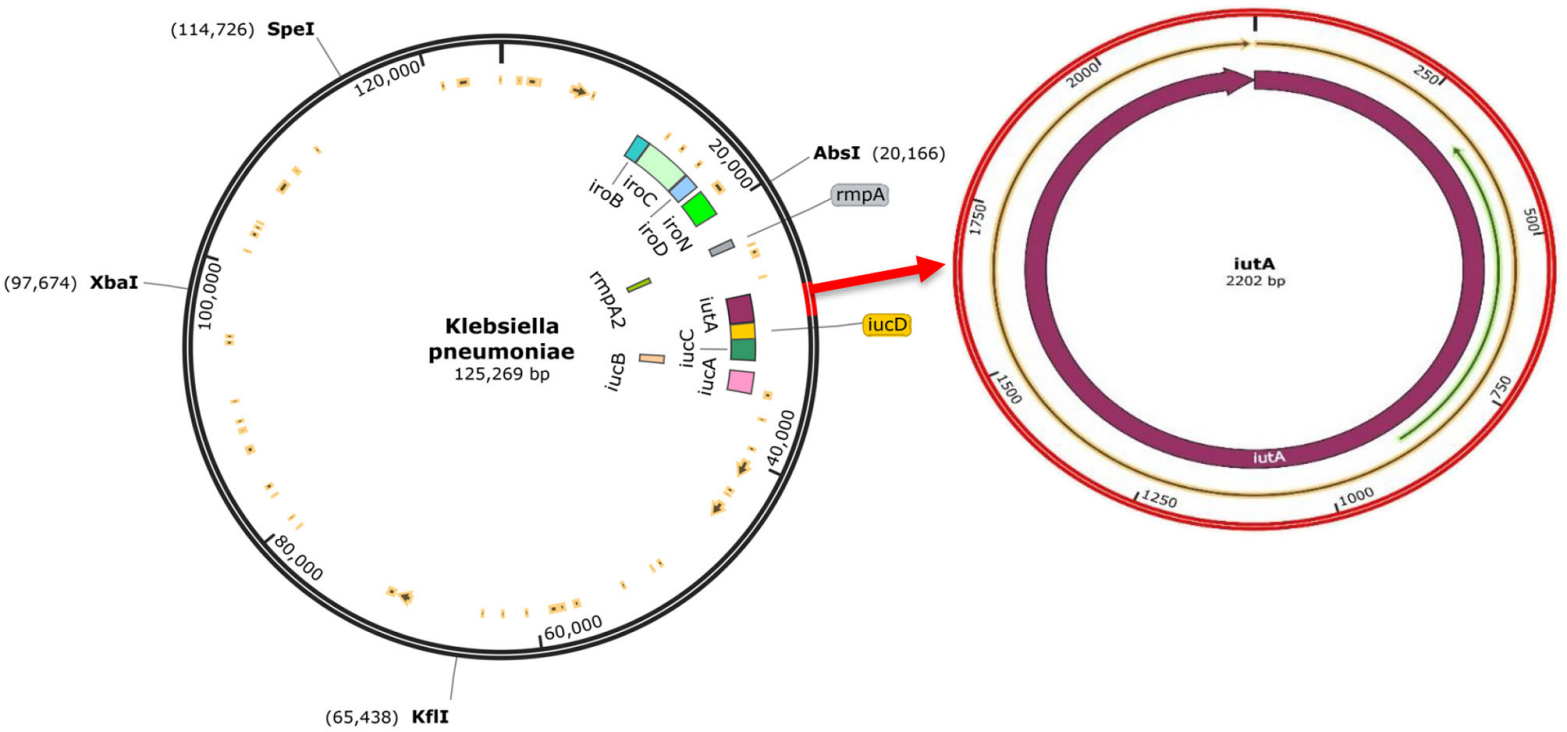
The complete sequence of *K.pneumoniae* plasmid (125,269 bp) showing the position of the hvKp-associated genes (*iucABCDiutA, iroBCDN, rmpA, and rmpA2*) adopted for the study **(left)**. The region of the iutA adopted as a biomarker in this study is amplified **(right)**, and corresponding Open Reading Frames (ORFs) are indicated on the prototype isolate. This visualization was generated using SnapGene software.

The analysis confirmed all isolates to be *K. pneumoniae*. The genome sequence revealed distinct virulence genes including siderophores (such as aerobactin, salmochelin, yersiniabactin, and enterobactin), iron uptake, allantoin metabolism, and colibactin with an identity range of 95%−100% (as shown in Supplementary Table 1). The prototype (Figure 4) shows *K. pneumoniae* plasmid (125,269 bp). The plasmid encodes *rmpA, rmpA2, iucABCD, iutA*, and *iroBCDN* amongst others.

**Figure 5:**
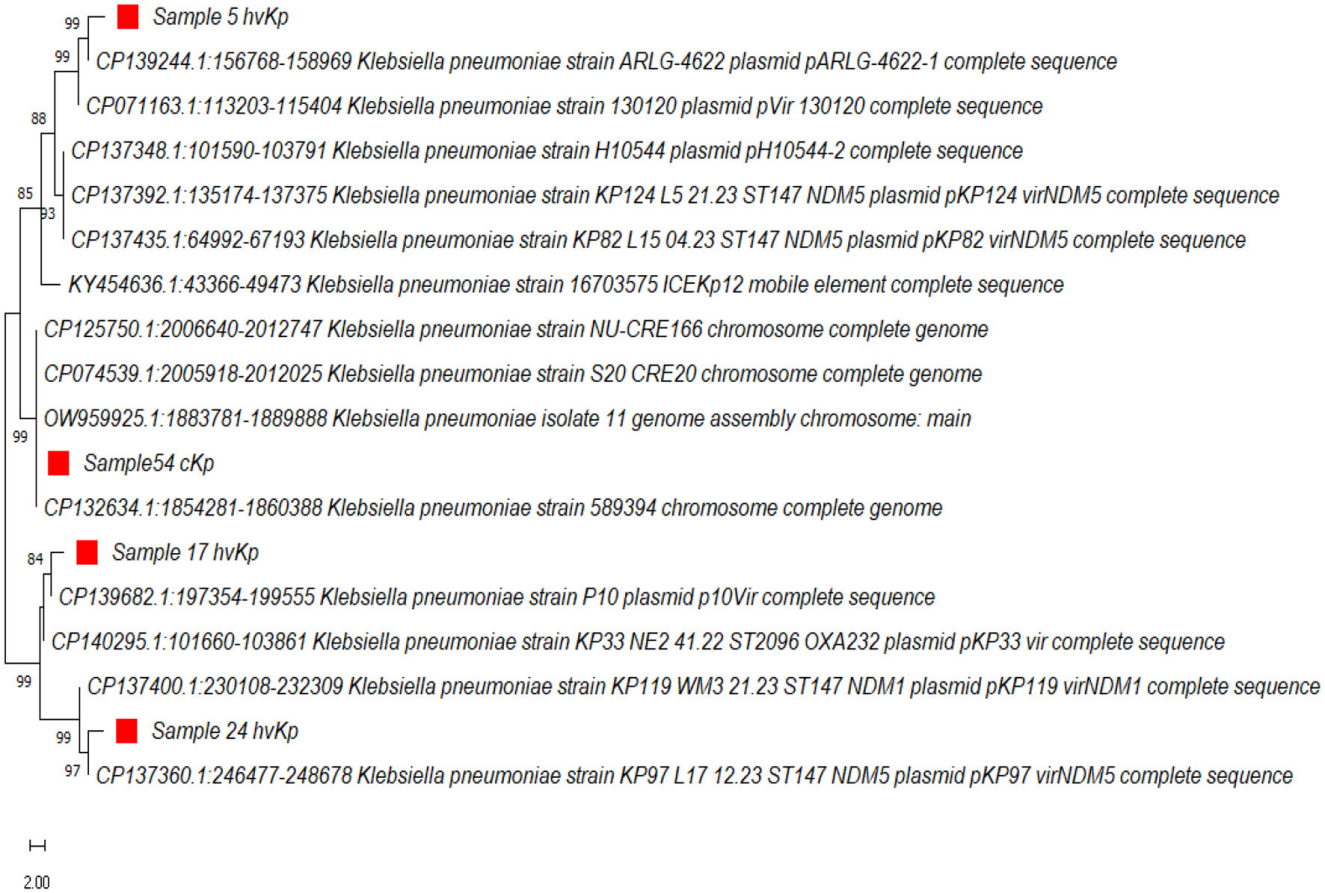
The evolutionary history was inferred using the Neighbor-Joining method (Saitou and Nei, 1987). The percentage of replicate trees in which the associated *K.pneumoniae* clustered together in the bootstrap test (1,000 replicates) are shown next to the branches (Felsenstein, 1985). The evolutionary distances were computed using the Maximum Composite Likelihood method and are in the units of the number of base substitutions per site. Evolutionary analyses were conducted in MEGA11 (Tamura et al., 2021).

The phylogenetic analysis of the curated sequences of *K. pneumoniae* further authenticated the virulence-associated factor of hvKp with >80% bootstrap values in the genes analyzed and their clustering patterns as shown in Figure 5, Sample 5 (hvKp) was found to cluster in the same clade with reference strain (Accession number: CP1392441) from the United States, having ~99% similarity, as well as reference strain (Accession number: CP071163) from West China. Also, sample 17 (hvKp) clustered unambiguously with other reference sequences; accession numbers: CP139682 and CP140295 from China and the United Kingdom respectively, while sample 24 was found to cluster with reference sequences CP137400 and CP137360 with 99% identity. Lastly, sample 54 (cKp) clustered unambiguously with other reference sequences (Accession number: CP124750, CP074539, OW969925, and CP132634) from NCBI with 98% similarity in one clade (Figure 5). The curated sequences from this study have been submitted to the GenBank and assigned with the following accession numbers: PP296963, PP296964, PP296965, and PP296966.

## 4 Discussion

This study aimed to investigate the occurrence of hvKp in Pelonomi Tertiary Hospital, one of the largest academic hospitals in the Free State Province. In addition, the aim was to assess the virulence of hvKp vs. cKp isolates and determine if any association between hypervirulent genetic determinates and virulence could be established. In total, 12.2% (*n* = 9/74) of the isolates collected over the 1-year period were hvKp. Based on available data, the majority of infections caused by these hvKp strains were community-acquired. These findings correlate with the literature which has documented a relationship between hypervirulence and community-acquired infections (CAIs) (Fang et al., 2007; Shankar et al., 2018; Wyres et al., 2020).

Several studies have described the importance of primary pyogenic liver abscess (PLA) or secondary infections (endophthalmitis and meningitis) as invasive infections in community-acquired hvKp patients (Fang et al., 2007; Guo et al., 2017; Li et al., 2021). This includes a study by Liu et al. (2014), who reported that hvKp strains found in the community caused severe infections such as liver abscess and pneumonia. Other studies reported that no statistically significant difference (*p* > 0.05) had been observed between hvKp and cKp regarding the severity of disease (Yan et al., 2016). Our findings support the latter, as no statistically significant association between hvKp/cKp and severity of disease was found. However, four of the nine hvKp patients had moderate to severe infections associated with organ impairment. In contrast, most patients with cKp infections had mild symptoms and were treated as outpatients. All hvKp isolates from this study were sensitive to antimicrobial drugs, except intrinsic resistance to penicillin and none (0/9) were extended-spectrum beta-lactamase (ESBL) producers.

The phenotypic, and genotypic differences between cKp and hvKp regarding virulence-associated genes are summarized in Table 4. The string test based on hypermucoviscous (hmv) phenotype (string ≥5 mm) has widely been used as a marker for hvKp, with ~90% predicted accuracy for clinical hvKp strains (Russo et al., 2018). However, this semi-qualitative method is easily influenced by colony conditions and the user's technique (Tan et al., 2014). The hmv phenotype from this study was found in 7/9 (78%) hvKp strains. An additional cKp (1/64) strain also possessed this phenotype. Similar results were reported by Zhang et al. (2016) who reported an average 75% (65/87) of hmv phenotype. Moreover, six hmv phenotypes were further detected in cKp strains and suggested that the hmv phenotype is indeed not a suitable biomarker for hypervirulence as some cKp strains also possesses this phenotype (Zhang et al., 2016).

The use of *iutA* as a biomarker in this current study confirmed that *iutA* is a reliable indication of hypervirulence (Hsieh et al., 2008; Hsu et al., 2011; Russo et al., 2014; Lam et al., 2018a,b). This is supported by current findings that the *iutA* gene was only detected in *K. pneumoniae* isolates that also possessed the virulent determinants *rmpA, iroB* and *rmpA2* (Russo et al., 2014; Russo and Marr, 2019). These three genetic determinants along with *iutA* are located on large virulence plasmids that cKp do not possess (Russo et al., 2014; Nahavandinejad and Asadpour, 2017; Russo and Marr, 2019; Choby et al., 2020). The results from the current study further showed that these genetic determinants are located on large virulence plasmids, and are associated with hypervirulence as none of these genetic determinants were found in isolate 54 (cKp) in this study, this is supported by the findings in a recent study conducted on hvKp from hospital and community settings that showed the presence of *iutA, iucB, iucC*, and *iutA* in all hvKp isolates (Raj et al., 2022). Recent studies have reported that cKp strains can acquire hvKp virulence-like plasmids that encode virulence-associated determinants (Gu et al., 2018; Huang et al., 2018; Russo and Marr, 2019). Therefore, more studies are needed to understand in depth the acquired virulence by cKp strains, e.g., through plasmids, and whether they result in enhanced virulence in the host.

Using the *C. elegans* nematode model, hvKp isolates were more virulent than cKp, in particularly isolate 1 (that contained all five hypervirulent genetic determinants including K1) and isolate 5 (which had five genes as well as K1 and K2). These findings are in keeping with Pomakova et al. (2012), who noted the presence of genetic determinants (K1/K2, *iroB* and *rmpA*) in hvKp strains (defined by positive hmv phenotype). Pomakova et al. (2012) further reported increased virulence in a rat abscess model when all hvKp genetic determinants were present. Other studies found that despite an isolate being hmv-negative, it still exhibited high virulence when possessing *rmpA* and *iucABCDiutA* genes (Li et al., 2019).

The presence of the hmv phenotype and *irp2* gene did not have any significant effect in virulence of *K. pneumoniae* strains. Isolate 24 which lacked the hmv phenotype was more virulent than isolate 17, which had the hmv phenotype (Figure 3B), while isolate 54 that possessed only the *irp2* was not significantly associated with a higher rate of killing *C. elegans*, as >60% of the worms survived by day 10. These results are in line with the literature, as various authors reported similar findings that these two factors could be found in cKp strains and therefore are not suitable markers for hypervirulence and not statistically significantly virulent in *in vivo* models (Wu et al., 2017; Lam et al., 2018a,b; Wyres et al., 2020).

A significant difference between isolate 17 (belonging to capsule type K2) and isolate 1 (belonging to K1) was noted, as isolate 1 was more virulent than isolate 17. Isolate 5 (belonged to K1) was also more virulent than isolate 17 (belonged to K2). Studies have shed some additional light on the virulence of these two capsule types. It is believed that the association of hvKp with K1/K2 serotypes depends on the presence of distinct sequence types (STs) that are conserved or diverse between the two serotypes (Russo et al., 2014; Lam et al., 2018a,b). The ST 23 is associated with the K1 capsule type, while distinct STs are associated with K2 (e.g., ST 65, ST 86, ST 375 and ST 380) (Follador et al., 2016; Catalán-Nájera et al., 2017; Martin and Bachman, 2018). Findings from multi-locus sequence typing (MLST) in this study support the above as it revealed that capsule K1 indeed belonged to ST23, while K2 belonged to ST 65. The capsule K1 serotype is more virulent compared to K2 (Russo and Marr, 2019). The *C. elegans* killing assay from the current study showed that isolate 5 (ST 23, K1) was more virulent than isolate 17 (ST 65, K2). Furthermore, a gene encoding an alginate lyase isozyme was exclusively found in strains of the K1 serotype, which was associated with enhanced virulence and co-infection in the host (Nahavandinejad and Asadpour, 2017). More genomic sequencing is still required to understand the genetic determinants and the association between the capsule type and invasive infections.

The phylogenetic analysis from this study showed a high percentage of relatedness (89%−99%) with other reference sequences from global endemic countries such as West China, United Kingdom, and the United States (Figure 5). Also, the clustering pattern of the cKp and hvKp from this study showed a 100% similarity of relatedness based on the evolutionary tree (Figure 5), owing to the indisputable ubiquitous nature and ability of contagious pathogens to cross populations.

This study had limitations, including a small sample size. Secondly, the clinical history of some patients was incomplete we were unable to establish the underlying conditions of patients.

In conclusion, to the best of our knowledge, this is the first study to confirm the presence of hvKp in one of the largest hospitals in an African developing country. The results from this study suggest that hvKp strains are more virulent relative to cKp in *in vivo* models which we considered as the strength of this study amongst others. Enhanced virulence is associated with the presence of virulence determinants, particularly K1/K2, *iroB* and *rmpA*. Currently, the occurrence of hvKp in the Free State Province is not a major concern, although further studies in different settings are recommended to deduce whether hypervirulent *Klebsiella pneumoniae* is a public concern as it is globally.

## Data availability statement

The data presented in the study are deposited in the GenBank repository, accession number: PP296963, PP296964, PP296965, and PP296966.

## Ethics statement

The studies involving humans were approved by Health Sciences Research Ethics Committee, University of the Free State. The studies were conducted in accordance with the local legislation and institutional requirements. The human samples used in this study were acquired from the National Health Laboratory Service (NHLS), Pelonomi Tertiary Hospital, in Bloemfontein, Free State Province, South Africa. Written informed consent for participation was not required from the participants or the participants' legal guardians/next of kin in accordance with the national legislation and institutional requirements. The manuscript presents research on animals that do not require ethical approval for their study.

## Author contributions

LD: Conceptualization, Data curation, Formal analysis, Investigation, Methodology, Visualization, Writing—original draft, Project administration, Writing—review & editing. OA: Conceptualization, Formal analysis, Visualization, Writing—review & editing. EA-C: Conceptualization, Formal analysis, Investigation, Writing—review & editing. CP: Formal analysis, Investigation, Writing—review & editing. NM: Formal analysis, Investigation, Writing—review & editing. MD: Conceptualization, Formal analysis, Writing—review & editing. JM: Conceptualization, Investigation, Supervision, Writing—review & editing.

## References

[B1] Brenner S. (1974). The genetics of *Caenorhabditis elegans*. Genetics 77, 73–94. 10.1093/genetics/77.1.71 4366476 PMC1213120

[B2] Catalán-Nájera J. C. Garza-Ramos U. Barrios-Camacho H. (2017). Hypervirulence and hypermucoviscosity: two different but complementary *Klebsiella* spp. phenotypes? Virulence 7, 1111–1123. 10.1080/21505594.2017.1317412 28402698 PMC5711391

[B3] Choby J. E. Howard-Anderson J. Weiss D. S. (2020). Hypervirulent *Klebsiella pneumoniae* – clinical and molecular perspectives. J. Intern. Med. 287, 283–300. 10.1111/joim.13007 31677303 PMC7057273

[B4] Compain F. Babosan A. Brisse S. Genel N. Audo J. Ailloud F.. (2014). Multiplex PCR for detection of seven virulence factors and K1/K2 capsular serotypes of *Klebsiella pneumoniae*. J. Clin. Microbiol. 52, 4377–4380. 10.1128/JCM.02316-14 25275000 PMC4313302

[B5] Conlan S. Kong H. H. Segre J. A. (2012). Species-level analysis of DNA sequence data from the NIH Human Microbiome Project. PLoS ONE 7:e47075. 10.1371/journal.pone.0047075 23071716 PMC3468466

[B6] Fang C. T. Lai S. Y. Yi W. C. Hsueh P. R. Liu K. L. Chang S. C.. (2007). *Klebsiella pneumoniae* genotype K1: an emerging pathogen that causes septic ocular or central nervous system complications from pyogenic liver abscess. Clin. Infect. Dis. 45, 284–293. 10.1086/519262 17599305

[B7] Felsenstein J. (1985). Confidence limits on phylogenies: an approach using the bootstrap. Evolution 39, 783–791. 10.1111/j.1558-5646.1985.tb00420.x 28561359

[B8] Follador R. Heinz E. Wyres K. L. Ellington M. J. Kowarik M. Holt K. E.. (2016). The diversity of *Klebsiella pneumoniae* surface polysaccharides. Microb. Genom. 2:e000073. 10.1099/mgen.0.000073 28348868 PMC5320592

[B9] Gu D. Dong N. Zheng Z. Lin D. Huang M. Wang L.. (2018). A fatal outbreak of ST11 carbapenem-resistant hypervirulent *Klebsiella pneumoniae* in a Chinese hospital: a molecular epidemiological study. Lancet Infect. Dis. 18, 37–46. 10.1016/S1473-3099(17)30489-9 28864030

[B10] Guo Y. Wang S. Zhan L. Jin Y. Duan J. Hao Z.. (2017). Microbiological and clinical characteristics of hypermucoviscous *Klebsiella pneumoniae* isolates associated with invasive infections in China. Front. Cell Infect. Microbiol. 7:24. 10.3389/fcimb.2017.00024 28203549 PMC5286779

[B11] Han S. H. (1995). Review of hepatic abscess from *Klebsiella pneumoniae* – an association with diabetes mellitus and septic endophthalmitis. West. J. Med. 162, 220–224. 7725704 PMC1022703

[B12] Han S. K. Lee D. Lee H. Kim D. Son H. G. Yang J. S.. (2016). OASIS 2: online application for survival analysis 2 with features for the analysis of maximal lifespan and healthspan in aging research. Oncotarget 7, 56147–56152. 10.18632/oncotarget.11269 27528229 PMC5302902

[B13] Hsieh P. F. Lin T. L. Lee C. Z. Tsai S. F. Wang J. T. (2008). Serum-induced iron-acquisition systems and TonB contribute to virulence in *Klebsiella pneumoniae* causing primary pyogenic liver abscess. J. Infect. Dis. 197, 1717–1727. 10.1086/588383 18433330

[B14] Hsu C. R. Lin T. L. Chen Y. C. Chou H. C. Wang J. T. (2011). The role of *Klebsiella pneumoniae rmpA* in capsular polysaccharide synthesis and virulence revisited. Microbiology 157, 3446–3457. 10.1099/mic.0.050336-0 21964731

[B15] Huang Y. H. Chou S. H. Liang S. W. Ni C. E. Lin Y. T. Huang Y. W.. (2018). Emergence of an XDR and carbapenemase-producing hypervirulent *Klebsiella pneumoniae* strain in Taiwan. J. Antimicrob. Chemother. 73, 2039–2046. 10.1093/jac/dky164 29800340

[B16] Hyun M. Lee J. Y. Ryu S. Y. Ryoo N. Kim H. A. (2019). Antibiotic resistance and clinical presentation of health care-associated hypervirulent *Klebsiella pneumoniae* infection in Korea. Microb. Drug Resist. 25, 1204–1209. 10.1089/mdr.2018.0423 31066617

[B17] Kamaladevi A. Balamurugan K. (2016). Lipopolysaccharide of *Klebsiella pneumoniae* attenuates immunity of *Caenorhabditis elegans* and evades by altering its supramolecular structure. Royal Soc. Chem. Adv. 6, 30070–30080. 10.1039/C5RA18206A

[B18] Karampatakis T. Tsergouli K. Behzadi P. (2023). Carbapenem-resistant *Klebsiella pneumoniae*: virulence factors, molecular epidemiology and latest updates in treatment options. Antibiotics 12:234. 10.3390/antibiotics12020234 36830145 PMC9952820

[B19] Ko W. Paterson D. L. Sagnimeni A. J. Hansen D. S. Von Gottberg A. Mohapatra S.. (2002). Community-acquired *Klebsiella pneumoniae* bacteremia : global differences in clinical patterns. Emerg. Infect. Dis. 8, 160–166. 10.3201/eid0802.010025 11897067 PMC2732457

[B20] Lam M. M. C. Wick R. R. Wyres K. L. Gorrie C. L. Judd L. M. Jenney A. W. J.. (2018a). Genetic diversity, mobilisation and spread of the yersiniabactin-encoding mobile element ICE*Kp* in *Klebsiella pneumoniae* populations. Microb. Genom. 4:e000196. 10.1099/mgen.0.000196 29985125 PMC6202445

[B21] Lam M. M. C. Wyres K. L. Judd L. M. Wick R. R. Jenney A. Brisse S.. (2018b). Tracking key virulence loci encoding aerobactin and salmochelin siderophore synthesis in *Klebsiella pneumoniae*. Genome Med. 10:77. 10.1186/s13073-018-0587-5 30371343 PMC6205773

[B22] Lan Y. Zhou M. Jian Z. Yan Q. Wang S. Liu W.. (2019). Prevalence of *pks* gene cluster and characteristics of *Klebsiella pneumoniae*-induced bloodstream infections. J. Clin. Lab. Anal. 33:e22838. 10.1002/jcla.22838 30737883 PMC6528554

[B23] Larsen M. V. Cosentino S. Rasmussen S. Friis C. Hasman H. Marvig R. L.. (2012). Multilocus sequence typing of total-genome-sequenced bacteria. J. Clin. Microbiol. 50, 1355–1361. 10.1128/JCM.06094-11 22238442 PMC3318499

[B24] Li G. Sun S. Zhao Z. Y. (2019). The pathogenicity of *rmpA* or aerobactin-positive *Klebsiella pneumoniae* in infected mice. J. Int. Med. Res. 47, 4346–4352. 10.1177/0300060519863544 31328596 PMC6753543

[B25] Li J. Ren J. Wang W. Wang G. Gu G. Wu X.. (2018). Risk factors and clinical outcomes of hypervirulent *Klebsiella pneumoniae* induced bloodstream infections. Eur. J. Clin. Microbiol. Infect. Dis. 37, 679–689. 10.1007/s10096-017-3160-z 29238932

[B26] Li Y. Li Z. Qian S. Dong F. Wang Q. Zhang P.. (2021). A fatal case of liver abscess caused by hypervirulent *Klebsiella pneumoniae* in a diabetic adolescent: a clinical and laboratory study. Pediatr. Investig. 5, 118–124. 10.1002/ped4.12238 34179708 PMC8212719

[B27] Liu C. Shi J. Guo J. (2018). High prevalence of hypervirulent *Klebsiella pneumoniae* infection in the genetic background of elderly patients in two teaching hospitals in China. Infect. Drug Resist. 11, 1031–1041. 10.2147/IDR.S161075 30104891 PMC6074765

[B28] Liu Y. M. Li B. B. Zhang Y. Y. Zhang W. Shen H. Li H.. (2014). Clinical and molecular characteristics of emerging hypervirulent *Klebsiella pneumoniae* bloodstream infections in mainland China. Antimicrob. Agents Chemother. 58, 5379–5385. 10.1128/AAC.02523-14 24982067 PMC4135864

[B29] Liu Z. Gu Y. Li X. Liu Y. Ye Y. Guan S. Li J. (2019). Identification and characterization of NDM-1-producing hypervirulent (hypermucoviscous) *Klebsiella pneumoniae* in China. Ann. Lab. Med. 39, 167–175. 10.3343/alm.2019.39.2.167 30430779 PMC6240523

[B30] Martin R. M. Bachman M. A. (2018). Colonization, infection, and the accessory genome of *Klebsiella pneumoniae*. Front. Cell. Infect. Microbiol. 8:4. 10.3389/fcimb.2018.00004 29404282 PMC5786545

[B31] Mukherjee S. Mitra S. Dutta S. Basu S. (2021). Neonatal sepsis: the impact of carbapenem-resistant and hypervirulent *Klebsiella pneumoniae*. Fron. Med. 8*:*634349. 10.3389/fmed.2021.634349 34179032 PMC8225938

[B32] Nahavandinejad M. Asadpour L. (2017). Mucoviscosity determination and detection of maga and rmpa genes in clinical isolates of *Klebsiella pneumoniae* in northern Iran. Crescent J. Med. Biol. Sci. 4, 104–107.

[B33] Paczosa M. K. Mecsas J. (2016). *Klebsiella pneumoniae*: going on the offense with a strong defense. Microbiol. Mol. Biol. Rev. 80, 629–661. 10.1128/MMBR.00078-15 27307579 PMC4981674

[B34] Pomakova D. K. Hsiao C. B. Beanan J. M. Olson R. MacDonald U. Keynan Y.. (2012). Clinical and phenotypic differences between classic and hypervirulent *Klebsiella pneumonia*: an emerging and under-recognized pathogenic variant. Eur. J. Clin. Microbiol. Infect. Dis. 31, 981–989. 10.1007/s10096-011-1396-6 21918907

[B35] Prjibelski A. Antipov D. Meleshko D. Lapidus A. Korobeynikov A. (2020). Using SPAdes de novo assembler. Curr. Protoc. Bioinformatics 70:e102. 10.1002/cpbi.102 32559359

[B36] Raj S. Sharma T. Pradhan D. Tyagi S. Gautam H. Singh H.. (2022). Comparative analysis of clinical and genomic characteristics of hypervirulent *Klebsiella pneumoniae* from hospital and community settings: experience from a tertiary healthcare center in India. Microbiol. Spectr. 10, e00376-22. 10.1128/spectrum.00376-22 36043878 PMC9602566

[B37] Remya P. Shanthi M. Sekar U. (2018). Occurance and characterization of hyperviscous K1 and K2 serotype in *Klebsiella pneumoniae*. J. Lab. Physicians 10, 283–288. 10.4103/JLP.JLP_48_18 30078963 PMC6052812

[B38] Russo T. Olson R. Fang C. T. Stoesser N. Miller M. MacDonald U.. (2018). Identification of biomarkers for differentiation of hypervirulent *Klebsiella pneumoniae* from classical *K. pneumoniae*. J. Clin. Microbiol. 56, e00776-18. 10.1128/JCM.00776-18 29925642 PMC6113484

[B39] Russo T. A. Marr C. M. (2019). Hypervirulent *Klebsiella pneumoniae*. Clin. Micriobiol. Rev. 32, e00001-19. 10.1128/CMR.00001-19 31092506 PMC6589860

[B40] Russo T. A. Olson R. MacDonald U. Beanan J. Davidson B. A. (2015). Aerobactin, but not yersiniabactin, salmochelin, or enterobactin, enables the growth/survival of hypervirulent (hypermucoviscous) *Klebsiella pneumoniae ex vivo* and *in vivo*. Infect. Immun. 83, 3325–3333. 10.1128/IAI.00430-15 26056379 PMC4496593

[B41] Russo T. A. Olson R. Macdonald U. Metzger D. Maltese L. M. Drake E. J.. (2014). Aerobactin mediates virulence and accounts for increased siderophore production under iron-limiting conditions by hypervirulent (hypermucoviscous) *Klebsiella pneumoniae*. Infect. Immun. 82, 2356–2367. 10.1128/IAI.01667-13 24664504 PMC4019165

[B42] Saitou N. Nei M. (1987). The neighbor-joining method: a new method for reconstructing phylogenetic trees. Mol. Biol. Evol. 4, 406–425. 3447015 10.1093/oxfordjournals.molbev.a040454

[B43] Shankar C. Veeraraghavan B. Nabarro L. E. B. Ravi R. Ragupathi N. K. D. Rupali P.. (2018). Whole genome analysis of hypervirulent *Klebsiella pneumoniae* isolates from community and hospital acquired bloodstream infection. BMC Microbiol. 18:6. 10.1186/s12866-017-1148-6 29433440 PMC5809863

[B44] Shon A. S. Bajwa R. P. S. Russo T. A. (2013). Hypervirulent (hypermucoviscous) *Klebsiella pneumoniae*: a new and dangerous breed. Virulence 4, 107–118. 10.4161/viru.22718 23302790 PMC3654609

[B45] Su C. Wu T. Meng B. Yue C. Sun Y. He L.. (2021). High prevalence of *Klebsiella pneumoniae* infections in AnHui Province: clinical characteristic and antimicrobial resistance. Infect. Drug Resist. 14, 5069–5078. 10.2147/IDR.S336451 34880632 PMC8645949

[B46] Tamura K. Stecher G. Kumar S. (2021). MEGA 11: molecular evolutionary genetics analysis version 11. Mol. Biol. Evol. 38, 3022–3027. 10.1093/molbev/msab120 33892491 PMC8233496

[B47] Tan T. Y. Cheng Y. Ong M. Ng L. S. (2014). Performance characteristics and clinical predictive value of the string test for detection of hepato-virulent *Klebsiella pneumoniae* isolated from blood cultures. Diagn. Microbiol. Infect. Dis. 78, 127–128. 10.1016/j.diagmicrobio.2013.10.014 24321354

[B48] Ullmann U. Podschun R. (1998). *Klebsiella* spp. as nosocomial pathogens: epidemiology, taxonomy, typing methods, and pathogenicity factors. Clin. Microbiol. Rev. 11, 589–603. 10.1128/CMR.11.4.589 9767057 PMC88898

[B49] Wang C. H. Lu P. L. Liu E. Y. Chen Y. Y. Lin F. M. Lin Y. T.. (2019). Rapid identification of capsular serotype K1/K2 *Klebsiella pneumoniae* in pus samples from liver abscess patients and positive blood culture samples from bacteremia cases via an immunochromatographic strip assay. Gut Pathog. 11:11. 10.1186/s13099-019-0285-x 30828389 PMC6385414

[B50] Wang J. H. Liu Y. C. Lee S. S. Yen M. Y. Chen Y. S. Wang J. H.. (1998). Primary liver abscess due to *Klebsiella pneumoniae* in Taiwan. Clin. Infect. Dis. 26, 1434–1438. 10.1086/516369 9636876

[B51] Wu H. Li D. Zhou H. Sun Y. Guo L. Shen D.. (2017). Bacteremia and other body site infection caused by hypervirulent and classic *Klebsiella pneumoniae*. Microb. Pathog. 104, 254–262. 10.1016/j.micpath.2017.01.049 28132768

[B52] Wyres K. L. Nguyen T. N. T. Lam M. M. C. Judd L. M. van Vinh Chau N. Dance D. A. B.. (2020). Genomic surveillance for hypervirulence and multi-drug resistance in invasive *Klebsiella pneumoniae* from South and Southeast Asia. Genome Med. 12:11. 10.1186/s13073-019-0706-y 31948471 PMC6966826

[B53] Wyres K. L. Wick R. R. Gorrie C. Jenney A. Follador R. Thomson N. R.. (2016). Identification of Klebsiella capsule synthesis loci from whole genome data. Microb. Genom. 2:e000102. 10.1099/mgen.0.000102 28348840 PMC5359410

[B54] Yan Q. Zhou M. Zou M. Liu W. E. (2016). Hypervirulent *Klebsiella pneumoniae* induced ventilator-associated pneumonia in mechanically ventilated patients in China. Eur. J. Clin. Microbiol. Infect. Dis. 35, 387–396. 10.1007/s10096-015-2551-2 26753990

[B55] Zhang R. Lin D. Chan E. W. Gu D. Chen G. X. Chen S.. (2016). Emergence of carbapenem-resistant serotype K1 hypervirulent *Klebsiella pneumoniae* strains in China. Antimicrob. Agents Chemother. 60, 709–711. 10.1128/AAC.02173-15 26574010 PMC4704206

