# Supplementary material for: Hypervirulent Klebsiella pneumoniae in a South African tertiary hospital—Clinical profile, genetic determinants, and virulence in Caenorhabditis elegans
Source: Front Microbiol. 2024 May 23;15:1385724. doi: 10.3389/fmicb.2024.1385724 (PMC11156222; doi:10.3389/fmicb.2024.1385724)
Supplement: Supplementary file 1 [file Table_1.docx]

**Supplemental Table 1.** List of *Klebsiella pneumoniae* virulence-associated genes and identity from 95%-100%

| **Gene** | **Identity (95–100%)** |
| --- | --- |
| *gcl_1* | 100 |
| *ybbW_1* | 100 |
| *allB_1* | 100 |
| *ybbY_1* | 100 |
| *ylbE_1* | 100 |
| *KP1_1364_1* | 100 |
| *allC_1* | 100 |
| *glxK_1* | 100 |
| *allD_1* | 100 |
| *allS_1* | 100 |
| *arcC_1* | 100 |
| *glxR_1* | 100 |
| *allR_1* | 100 |
| *ylbF_1* | 100 |
| *KP1_1371_1* | 100 |
| *hyi_1* | 100 |
| *allA_1* | 100 |
| *mceG_1* | 100 |
| *mceJ_1* | 100 |
| *mceH_1* | 100 |
| *mceD_1* | 100 |
| *mceC_1* | 100 |
| *mceI_1* | 100 |
| *mceE_1* | 100 |
| *mceA_1* | 100 |
| *mceB_1* | 100 |
| *kfuB_1* | 100 |
| *kfuC_1* | 100 |
| *kfuA_1* | 100 |
| *ylbE_1* | 98,73015873 |
| *ybbW_1* | 95,18900344 |
| *KP1_1371_1* | 100,2554278 |
| *iroC_1* | 95,65217391 |
| *iroN_1* | 97,1954023 |
| *clbB_1* | 100 |
| *clbH_1* | 100 |
| *clbI_1* | 100 |
| *clbC_1* | 100 |
| *clbG_1* | 100 |
| *clbF_1* | 100 |
| *clbD_1* | 100 |
| *clbA_1* | 100 |
| *clbE_1* | 100 |
| *clbR_1* | 100 |
| *mrkD_1* | 100 |
| *mrkJ_1* | 100 |
| *mrkH_1* | 100 |
| *mrkB_1* | 100 |
| *mrkF_1* | 100 |
| *mrkA_1* | 100 |
| *mrkI_1* | 100 |
| *clbN_1* | 100 |
| *clbO_1* | 100 |
| *clbP_1* | 100 |
| *clbL_1* | 100 |
| *clbM_1* | 100 |
| *clbQ_1* | 100 |
| *iutA_1* | 100 |
| *iucC_1* | 100 |
| *iucA_1* | 100 |
| *iucD_1* | 100 |
| *iucB_1* | 100 |
| *rmpA2_1* | 100 |
| *rmpA_1* | 98,58044164 |
| *iroC_1* | 100 |
| *iroN_1* | 100 |
| *iroD_1* | 100 |
| *iroB_1* | 100 |
| *rmpA_1* | 100 |
| *rmpA2_1* | 97,65258216 |
| *ybtT_1* | 100 |
| *irp1_1* | 100 |
| *hyi_1* | 100 |
| *allA_1* | 100 |
| *glxK_1* | 100 |
| *ybtQ_1* | 100 |
| *ybtP_1* | 100 |
| *ybtE_1* | 100 |
| *ybtS_1* | 100 |
| *ybtX_1* | 100 |
| *ybbY_1* | 100 |
| *ylbE_1* | 100 |
