# Supplementary material for: Hypervirulent Klebsiella pneumoniae in a South African tertiary hospital—Clinical profile, genetic determinants, and virulence in Caenorhabditis elegans
Source: Front Microbiol. 2024 May 23;15:1385724. doi: 10.3389/fmicb.2024.1385724 (PMC11156222; doi:10.3389/fmicb.2024.1385724)
Supplement: Supplementary file 2 [file Table_2.docx]

| **Isolate number** | **spectrum of disease** | **Amakicin** | **Ampicillin** | Amoxicillin  **/Clavulanic acid** | **Cefuroxime** | Cefotaxime/  **Ceftriaxone** | **Gentamicin** | **Ciprofloxacin** | **Nitrofurantoin** | Trimethoprim/  **Sulfamethoxazole** | **Ertapenem** | **Imipenem** | **Meropenem** | **Pip-taz** |
| --- | --- | --- | --- | --- | --- | --- | --- | --- | --- | --- | --- | --- | --- | --- |
| 3 | severe | S | R | R | S | S | S | S | N | S | S | S | S | R |
| 4 | mild | S | R | S | S | S | S | S | S | S | S | S | S | S |
| 6 | mild | S | R | S | S | S | S | S | I | S | S | S | S | S |
| 7 | severe | S | R | S | S | S | S | S | I | S | S | S | S | S |
| 9 | severe | S | R | S | S | S | S | S | R | S | S | S | S | S |
| 10 | mild | S | R | S | S | S | S | S | S | S | S | S | S | S |
| 11 | severe | S | R | R | R | R | R | S | R | R | S | S | S | R |
| 12 | moderate | S | R | S | S | S | S | S | I | S | S | S | S | S |
| 13 | severe | S | R | S | S | S | S | S | I | S | S | S | S | S |
| 15 | mild | S | R | S | S | S | S | S | R | N | S | N | S | I |
| 16 | severe | S | R | S | S | S | S | S | I | S | S | S | S | S |
| 18 | mild | S | R | S | S | S | R | R | S | R | S | S | S | S |
| 19 | mild | S | R | S | S | S | S | S | I | S | S | S | S | I |
| 20 | mild | S | R | S | S | S | S | S | I | S | S | S | S | S |
| 21 | mild | S | R | S | S | S | S | S | I | R | S | S | S | S |
| 22 | mild | S | R | S | S | S | S | S | S | S | S | S | S | S |
| 23 | mild | S | R | S | S | S | S | S | I | S | S | S | S | S |
| 25 | mild | S | R | S | S | S | S | S | R | S | S |  | S | S |
| 26 | mild | S | R | R | R | R | R | S | R | R | S | S | S | I |
| 27 | severe | S | R | S | S | S | S | S | S | S | S | S | S | S |
| 28 | mild | S | R | S | S | S | S | S | R | S | S | S | S | S |
| 29 | mild | S | R | R | R | R | R | R | I | R | S | S | S | I |
| 30 | mild | S | R | S | S | S | S | S | S | S | S | S | S | S |
| 31 | severe | S | R | S | S | S | S | S | S | S | S | S | S | S |
| 32 | mild | S | R | S | S | S | S | S | I | S | S | S | S | S |
| 33 | severe | S | R | S | S | S | S | S | I | S | S | S | S | S |
| 34 | severe | S | R | S | S | S | S | S | I | S | S | S | S | S |
| 35 | mild | S | R | S | S | S | S | S | I | R | S | S | S | S |
| 36 | severe | S | R | S | S | S | S | S | N | S | S | S | S | S |
| 37 | severe | S | R | S | S | S | S | S | S | S | S | S | S | S |
| 39 | severe | S | R | R | R | R | R | S | R | R | S | S | S | R |
| 41 | mild | S | R | S | S | S | S | S | R | S | S | S | S | S |
| 42 | severe | S | R | S | S | S | S | S | I | S | S | S | S | S |
| 44 | moderate | S | R | S | R | R | R | S | I | R | S | S | S | S |
| 45 | mild | S | R | S | S | S | S | S | N | S | S | S | S | S |
| 47 | mild | S | R | S | S | S | S | S | R | S | S | S | S | I |
| 48 | mild | S | R | S | S | S | S | S | N | S | S | S | S | S |
| 49 | moderate | S | R | R | R | R | R | R | N | R | R | S | S | R |
| 50 | mild | S | R | S | S | S | S | S | N | S | S | S | S | S |
|  |  |  |  |  |  |  |  |  |  |  |  |  |  |  |
| 52 | moderate | S | R | R | R | R | R | R | N | R | R | S | S | R |
| 53 | * | S | R | S | S | S | S | S | S | S | S | S | S | S |
| 54 | severe | S | R | S | S | S | S | S | I | S | S | S | S | S |
| 55 | * | S | R | S | S | S | S | S | I | S | S | S | S | S |
| 56 | severe | S | R | S | S | S | S | S | S | S | S | S | S | S |
| 57 | mild | S | R | R | S | S | S | S | R | R | S | S | S | I |
| 58 | * | S | R | S | S | S | S | S | I | S | S | S | S | S |
| 59 | * | S | R | S | S | S | S | S | I | S | S | S | S | S |
| 60 | mild | S | R | S | S | S | S | S | I | S | S | S | S | S |
| 61 | * | S | R | S | S | S | S | S | I | R | S | S | S | S |
| 62 | * | S | R | S | S | S | S | S | S | S | S | S | S | S |
| 63 | * | S | R | R | R | R | R | R | R | R | I | S | S | R |
| 64 | mild | S | R | I | R | R | R | R | R | R | S | S | S | S |
| 65 | * | S | R | S | S | S | S | S | I | S | S | S | S | S |
| 66 | * | S | R | S | S | S | S | S | I | S | S | S | S | S |
| 67 | * | S | R | R | R | R | R | R | S | S | S | S | S | I |
| 68 | * | S | R | S | R | R | R | S | I | R | S | S | S | S |
| 69 | * | S | R | S | S | S | S | S | S | R | S | S | S | S |
| 70 | * | S | R | S | R | R | R | S | I | R | S | S | S | S |
| 71 | * | S | R | S | S | S | S | S | I | S | S | S | S | S |
| 72 | * | S | R | S | S | S | S | S | I | S | S | S | S | I |
| 74 | * | S | R | S | S | S | S | S | S | S | S | S | S | S |
| 75 | * | S | R | S | R | R | S | S | I | R | S | S | S | S |
| 76 | * | S | R | S | S | S | S | S | R | R | S | S | S | S |
| 77 | * | S | R | S | S | S | S | S | I | R | S | S | S | S |
| 78 | * | S | R | S | R | R | R | R | S | R | S | S | S | S |
| 79 | * | S | R | S | S | S | S | S | S | S | S | S | S | S |

**Keys**

S- Sensitive

R- Resistant

I-Intermediate

*- Not Known
