# Supplementary material for: Hypervirulent Klebsiella pneumoniae in a South African tertiary hospital—Clinical profile, genetic determinants, and virulence in Caenorhabditis elegans
Source: Front Microbiol. 2024 May 23;15:1385724. doi: 10.3389/fmicb.2024.1385724 (PMC11156222; doi:10.3389/fmicb.2024.1385724)
Supplement: Supplementary file 3 [file Image_1.pdf]

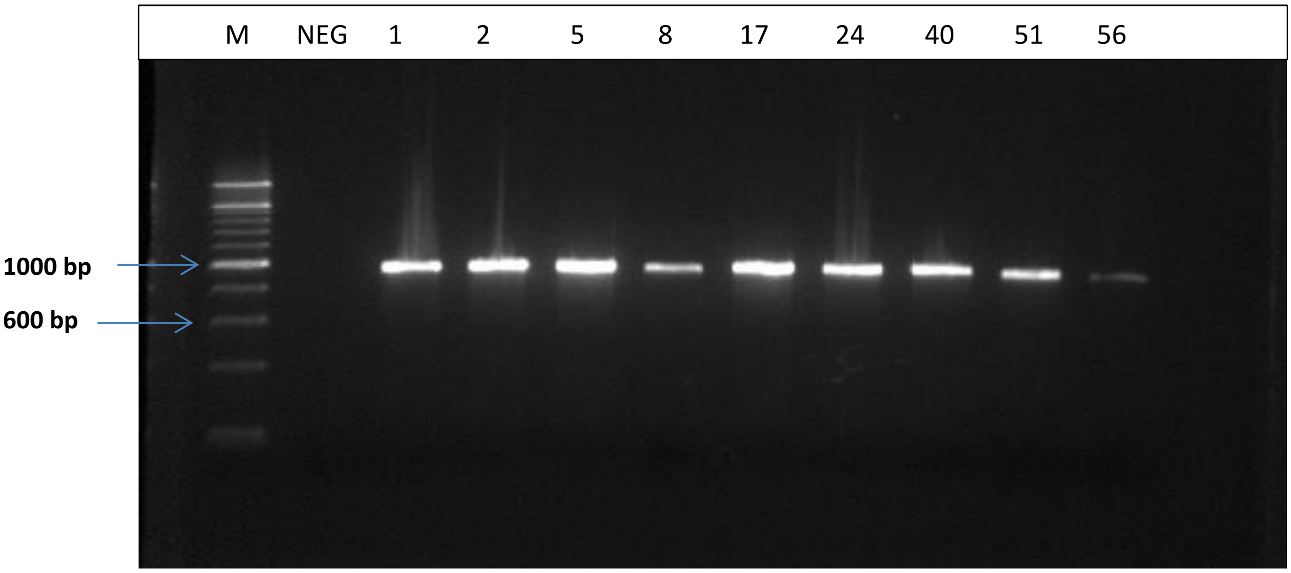

Supplementary figure 1: A visual representation of *iutA PCR* ran on 1.5% agarose gel electrophoresis. The first lane depicts a 200bp marker, lane 2 is a negative, and lanes 3-11 are hypervirulent *K. pneumoniae* amplicons.
